# The role of formyl peptide receptor 1 (FPR1) in neuroblastoma tumorigenesis

**DOI:** 10.1186/s12885-016-2545-1

**Published:** 2016-07-18

**Authors:** Igor Snapkov, Carl Otto Öqvist, Yngve Figenschau, Per Kogner, John Inge Johnsen, Baldur Sveinbjørnsson

**Affiliations:** Molecular Inflammation Research Group, Department of Medical Biology, Faculty of Health Sciences, University of Tromsø, Tromsø, Norway; Childhood Cancer Research Unit, Department of Women’s and Children’s Health, Karolinska Institutet, Stockholm, Sweden; Endocrinology Research Group, Department of Clinical Medicine, Faculty of Health Sciences, University of Tromsø, Tromsø, Norway; Department of Medical Biology, Faculty of Health Sciences, University of Tromsø, Tromsø, Norway; Department of Laboratory Medicine, University Hospital of North Norway, Tromsø, Norway

**Keywords:** FPR1, Formyl peptide receptor 1, Neuroblastoma, Inflammation

## Abstract

**Background:**

Formyl peptide receptor 1 (FPR1) is a G protein-coupled receptor mainly expressed by the cells of myeloid origin, where it mediates the innate immune response to bacterial formylated peptides. High expression of FPR1 has been detected in various cancers but the function of FPR1 in tumorigenesis is poorly understood.

**Methods:**

Expression of FPR1 in neuroblastoma cell lines and primary tumors was studied using RT-PCR, western blotting, immunofluorescence and immunohistochemistry. Calcium mobilization assays and western blots with phospho-specific antibodies were used to assess the functional activity of FPR1 in neuroblastoma. The tumorigenic capacity of FPR1 was assessed by xenografting of neuroblastoma cells expressing inducible FPR1 shRNA, FPR1 cDNA or control shRNA in nude mice.

**Results:**

FPR1 is expressed in neuroblastoma primary tumors and cell lines. High expression of FPR1 corresponds with high-risk disease and poor patient survival. Stimulation of FPR1 in neuroblastoma cells using fMLP, a selective FPR1 agonist, induced intracellular calcium mobilization and activation of MAPK/Erk, PI3K/Akt and P38-MAPK signal transduction pathways that were inhibited by using Cyclosporin H, a selective receptor antagonist for FPR1. shRNA knock-down of FPR1 in neuroblastoma cells conferred a delayed xenograft tumor development in nude mice, whereas an ectopic overexpression of FPR1 promoted augmented tumorigenesis in nude mice.

**Conclusion:**

Our data demonstrate that FPR1 is involved in neuroblastoma development and could represent a therapy option for the treatment of neuroblastoma.

**Electronic supplementary material:**

The online version of this article (doi:10.1186/s12885-016-2545-1) contains supplementary material, which is available to authorized users.

## Background

Neuroblastoma (NB) is a tumor of the peripheral nervous system, and is a common and deadly childhood tumor [1]. Despite extensive treatment, including chemotherapy, surgery, radiation therapy and immunotherapy, the survival rate among high-risk patients is less than 50 % [2, 3]. Hence, there is a great need for new therapies, particularly those based on a biological understanding of tumor development.

N-formyl peptide receptors, of which three have been described in humans (FPR1, FPR2 and FPR3), are involved in the regulation of innate immune responses [4]. FPRs are seven-transmembrane G protein-coupled receptors that were originally described in immune cells, and are important for the induction of inflammation and immune cell activation in response to N-formyl peptides produced by bacteria during infections [5]. FPR expression was subsequently shown in non-hematopoietic cells and tissues where the receptors are involved in tissue regeneration and wound healing [5]. Several studies have suggested a role for FPRs in the progression of tumors of different origin [6–11]. The involvement of FPRs in tumorigenesis seems to be context-specific. For instance, in gastric cancer a high expression of FPR1was associated with advanced disease and poor survival [11], while another study reported FPR1 to be a tumor suppressor by inhibition of angiogenesis [12]. In glioma, FPR was shown to mediate tumor cell chemotaxis, proliferation and the stimulation of angiogenesis, and to also induce an invasive phenotype, whereas in melanoma FPR promoted NK cell migration and inhibited tumor growth [6, 9, 13, 14].

In this study, we investigated the functional role of FPR1 in neuroblastoma development, and showed that high FPR1 expression is associated with high-risk disease and is important for neuroblastoma tumorigenesis.

## Methods

### Bioinformatics

FPR1 gene expression data were downloaded from R2: Genomics Analysis and Visualization Platform [15] (http://r2.amc.nl), and the R2 web-based application was used to generate Kaplan-Meier survival curves.

### Cell lines and reagents

The human neuroblastoma cell lines (SK-N-FI, SHEP-1, SK-N-BE (2), SK-N-SH, SK-N-AS, SH-SY5Y, SK-N-DZ) were purchased from the American Type Culture Collection (ATCC) (Table 1). The cells were cultured in humidified air with 5 % CO_2_ at 37 °C in RPMI-1640 medium with L-glutamine and sodium bicarbonate (R8758, Sigma-Aldrich Chemie GmbH, Steinheim, Germany), and supplemented with 10 % FBS. N-Formyl-L-methionyl-L-leucyl-L-phenylalanine (fMLP) was purchased from Sigma-Aldrich (F3506, St. Louis, MO, USA) and Cyclosporin H from Enzo Life Sciences (ALX-380-286, Farmingdale, NY, USA).

**Table 1:** Characteristics of the cell lines used in this study

	SK-N-FI	SHEP-1	SK-N-BE(2)	SK-N-SH	SK-N-AS	SH-SY5Y	SK-N-DZ
MYCN amplification	**-**	**-**	**+**	**-**	**-**	**-**	**+**
MDR phenotype	**+**	**-**	**+**	**+**	**+**	**-**	**-**
1p deletion	**-**	**-**	**+**	**-**	**+**	**-**	**-**
11q deletion	**-**	**-**	**-**	**-**	**+**	**-**	**+**

*MDR* multiple drug resistance

### Human tissue samples

Primary neuroblastoma samples were obtained during surgery, snap-frozen in liquid nitrogen and transferred to −80 °C for future analysis. Twenty-seven neuroblastoma samples derived from children of different ages and all clinical stages, including different biological subsets (MYCN amplification, 7 of 27; 1 p deletion, 9 of 27) were analyzed. Ethical approval was obtained from the Karolinska University Hospital Research Ethics Committee.

### Western blot analysis

Homogenized tissue specimens and cells were lysed directly in a RIPA lysis buffer (89901, Pierce Biotechnology, Rockford, IL, USA) with protease inhibitor cocktail (04693124001, Roche Applied Science, Basel, Switzerland) and Halt phosphatase inhibitor cocktail (78420, Pierce Biotechnology). Moreover, a mixture containing the NuPAGE LDS Sample Buffer (NP0008, Life Technologies, Carlsbad, CA, USA), NuPAGE Sample Reducing Agent (NP0004, Life Technologies) and distilled water were added to lysates. The samples were heated to 70 °C for 10 min, and equal amounts of protein were loaded into NuPAGE Novex 4-12 % Bis-Tris gel (NP0335PK, Life Technologies). Gel electrophoresis and blotting onto PVDF membrane (LC2005, Life Technologies) were performed according to the NuPAGE Technical Guide (Invitrogen). Tris buffered saline with 0.1 % Tween-20 (93773, Sigma) and 5 % Bio-Rad Blotting-grade blocker (170–6404, Hercules, CA, USA) were used for blocking, while primary and secondary antibodies were diluted in the blocking buffer. Membranes were probed with antibodies against FPR1 (ab113531, Abcam, Cambridge, UK), p44/42 MAPK (Erk1/2) (4695, Cell Signaling Technology, Danvers, MA, USA), Phospho-p44/42 MAPK (Erk1/2) (4370, Cell Signaling Technology, Danvers, MA), Akt (9272, Cell Signaling Technology), Phospho-Akt (9271, Cell Signaling Technology), P38-MAPK (9212, Cell Signaling Technology) and Phospho-P38-MAPK (9211, Cell Signaling Technology). Concentrations of the antibodies were 1:500, 1:1500, 1:2000, 1:1000, 1:1000, 1:1000 and 1:1000, respectively. Anti-beta Actin antibody (ab8227, Abcam) in a dilution of 1:5000 was used as a loading control. Goat Anti-Rabbit IgG H&L (HRP) antibodies (ab6721, Abcam) in a dilution of 1:5000 served as secondary antibodies. SuperSignal West Pico Chemiluminescent Substrate (34080 F, Pierce Biotechnology) was used for detection, and images were acquired on a Fujifilm LAS-3000 Imager.

### Reverse transcription PCR

Total RNA was isolated from pelleted cells and tumor tissue using the Qiagen RNeasy Mini Kit (74104, Hilden, Germany) according to the manufacturer’s protocol. The quantity and quality of the extracted RNA was determined with the use of a spectrophotometer NanoDrop ND-1000 (Wilmington, DE, USA). cDNA was synthesized from 1 μg of total RNA using the QuantiTect Reverse Transcription Kit (205310, Hilden) according to the manufacturer’s protocol. PCR was performed in a 25 μl reaction mixture containing 2 μl of cDNA (from isolated RNA), 12.5 μl of the JumpStart REDTaq® Ready Mix (Sigma-Aldrich, P0982), 0.5 μl of 10 μM forward and reverse primers, and 9.5 μl of ddH2O. The reactions were performed in a BioRad T-100 thermal cycler with the following conditions: initial denaturation at 95 °C for 2 min, denaturation at 95 °C for 30 s, annealing at 58 °C for 30 s and extension at 72 °C for 2 min. In total, 35 cycles were conducted with a final extension at 72 °C for 10 min. PCR products were electrophoresed on 2 % agarose gel and visualized under UV light. The sequences of PCR primers were FPR1 forward: 5′-TGG GAG GAC ATT GGC CTT TC-3′; and reverse: 5′-GGA TGC AGG ACG CAA ACA C-3′ (PrimerBank ID 36951095c1, http://pga.mgh.harvard.edu/primerbank/index.html); β-actin forward: 5′-CTC GAC ACC AGG GCG TTA T-3′; and reverse: 5′-CCA CTC CAT GCT CGA TAG GAT-3′.

### Immunohistochemistry

Formalin-fixed and paraffin-embedded tissue sections were deparaffinized in xylene and graded alcohols, hydrated and washed in a phosphate-buffered saline (PBS). After antigen retrieval in a sodium citrate buffer (pH 6) in a microwave oven, the endogenous peroxidase was blocked by 0.3 % H_2_O_2_ for 15 min. Sections were incubated overnight at 4 °C with a primary antibody (ab113531, Abcam). As a secondary antibody, the anti-rabbit-horseradish peroxidase (HRP) SuperPicTure Polymer detection kit was used (87–9663, Zymed-Invitrogen, San Francisco, CA, USA). A matched isotype control was also used as a control for nonspecific background staining.

### Immunofluorescence

For immunofluorescence studies, cells were grown on fibronectin coated chamber slides (Nunc, Roskilde, Denmark) for 24 h. Cultures were then washed and fixed with 2 % paraformaldehyde for 30 min. After washing with a PBS buffer, rabbit anti-FPR1 antibodies (described above) were incubated with cultures overnight at 4 °C. After rinsing in PBS, cultures were incubated with goat anti-rabbit IgG secondary antibodies conjugated with Alexa 488 (A-11008, Life Technologies, Eugene, OR, USA).

### Calcium mobilization assay

SK-N-SH cells (2.5x10^4^ cell per well) were seeded into an 8 well μ-Slide (80826, iBidi GmbH, Munich, Germany) and incubated overnight in a complete growth medium. The next day the cells were washed and preloaded with 20 μM Cal-520 (21131, AATBio, Sunnyvale, CA, USA) in HHBS with 0.04 % Pluronic® F-127 (20053, AATBio). After 90 min of incubation at 37 °C, a dye solution was replaced with HHBS and cells were subsequently examined with a Leica TCS SP5 confocal microscope. Before the addition of 10 nM of fMLP, a baseline was measured. Images were then obtained and analyzed using the Leica LAS AF software.

### Generation of neuroblastoma cells with a differential expression of FPR1

A plasmid containing a human sequence-verified FPR1 cDNA (Clone ID 3829614) was purchased from Thermo Fisher Scientific, as well as lentiviral vectors pTRIPZ_control with a scrambled shRNA (Clone ID RHS4743) and four different pTripz shRNA clones targeting FPR1 (Clone Ids V3THS_390191, V3THS_390189, V3THS_413003, V3THS_113957). Lentiviral helper packaging plasmid psPAX2 (Plasmid ID 12260) and envelope plasmid pMD2.G (Plasmid ID 12259) were purchased from Addgene.

All primers used were purchased from Integrated DNA technologies (IDT), with the exception of the RFP_2A_rev primer, which was purchased from Sigma.

LentiX HEK-293 T cells were purchased from Clonetech (632180, Mountain View, CA, USA) and grown at 37 °C and 5 % CO_2_ in Dulbecco’s Modified Eagle Medium (11966–025, Gibco™, Paisley, UK), supplemented with 10 % FBS; 100 μg/mL of streptomycin and 100 U/mL of penicillin. SK-N-AS cells were ordered from the American Type Culture Collection (ATCC) (CRL-2137) and grown at 37 °C and 5 % CO_2_ in a RPMI-1640 medium (21875–034, Gibco™) supplemented with 10 % FBS, 100 μg/mL of streptomycin and 100 U/mL of penicillin.

A lentiviral plasmid expressing turbo red fluorescent protein (tRFP) and FPR1 cDNA separated by a 2A sequence (pTripz_RFP_2A_FPR1) was constructed by amplification of a tRFP fragment from a 10 ng pTripz_control plasmid template using forward primer RFP_fwd (CGT TTA GTG AAC CGT CAG ATC GCA CCG GTC GCC ACC ATG AG) and reverse primer RFP_2A_rev (GTC TCC TGC TTG CTT TAA TAG AGA GAA GTT AGT AGC TCC AGA TCC TCT GTG CCC CAG TTT GCT A). FPR1 cDNA was amplified from a cDNA plasmid template (1 μl of glycerol bacterial stock) using a PAGE purified forward primer 2A_FPR1_fwd (GCT ACT AAC TTC TCT CTA TTA AAG CAA GCA GGA GAC GTG GAA GAA AAC CCA GGT CCT ATG GAG ACA AAT TCC TCT CTC CC) and reverse primer FPR1_rev (GCG GAG GCC ACG CGT CTA CTT TGC CTG TAA CTC CAC C). PTripz_control plasmid was cleaved using restriction enzymes AgeI_HF (R3552S, Ipswich, MA, USA) and MluI (R0198S) purchased from New England Biolabs (NEB), and gel purified using a 1 % TAE agarose gel. 100 ng of plasmid backbone was mixed with the PCR-generated tRFP and FPR1 cDNA fragments in equimolar amounts and ligated using 2X Gibson mix (E2611S, NEB). A 2 μl ligation mixture was used to transfect XL10 gold ultracompetent cells (200314, NEB) and spread on 100 μM Amp agar plates. The resultant colonies were picked, amplified and purified using alkaline lysis and precipitated using 10 % PEG-6000. All plasmids were sequence verified prior to transfection.

Lipofectamine LTX with plus reagent (15338100) was purchased from Life Technologies. Approximately 1 million LentiX Hek293T cells were resuspended in 5 ml of Opti-MEM® (31985–047, Gibco™). Plasmid DNA (1 μg of psPAX2, 1 μg of shRNA or control plasmid and 0.5 μg of pMD2.G) were mixed with 2.5 μl plus reagent and incubated for 5 min at room temperature. Next, 7.5 μl of lipofectamine LTX was added and the samples were incubated for an additional 30 min at RT before being added to the cells. Approximately 48 h post-transfection, the cell culture media was removed using a 5 ml syringe, filtered through 0.45 μm SUPOR syringe filters (4654, Ann Arbor, MI, USA) ordered from the Pall Corporation and stored at −80 °C.

Approximately 100,000 SK-N-AS cells were seeded per well in a 24-well plate in 1 ml RPMI-1640 medium supplemented with 10 % FBS, a penicillin/streptomycin solution and L-glutamine. Different amounts of virus-containing filtered Opti-MEM® were added to the cells (200 μl, 50 μl and 12.5 μl), and the cells were incubated at 37 °C for 48 h. After the recovery period, the infected cells were selected by adding puromycine to the complete RPMI-1640 medium at a final concentration of 0.5 μg/ml. In order to minimize off-target effects due to multiple integration, only wells that contained less than a few hundred CFU (colony forming units) were used.

### Xenograft experiment

In vivo xenograft studies were performed using 10 immunodeficient NMRI nu/nu mice (Taconic, Ejby, Denmark) per construct. Mice were subcutaneously injected in the flank with 0.9 x 10^6^ cells in 100 μl RPMI-1640. The mice received doxycycline in the water at a concentration of 1 μg/ml. Tumors were measured three times per week with digital calipers and the volume was calculated by the formula width^2^ × length × 0.44. The animals were sacrificed after they developed a tumor volume ≥ 1 cm^3^. The excised tumors were divided into two parts, which were then formalin-fixed and snap frozen. The animal experiments were approved by the Regional Ethics Committee (Stockholm Northern Board) for Animal Research (approval ID: N391/11), appointed and put under the control of the Swedish Board of Agriculture and the Swedish Court. The animal experiments presented herein were in accordance with the proper national regulations (SFS 1988:534, SFS 1988:539, and SFS 1988:541).

### Statistical analysis

Statistical analysis and Kaplan-Meier curves were prepared using a SigmaPlot version 13.0 (Systat Software, San Jose, CA, USA). Experimental groups were compared using a log-rank test.

## Results

### High-level expression of FPR1 is a negative prognostic factor for neuroblastoma patient survival

**Fig. 1:**
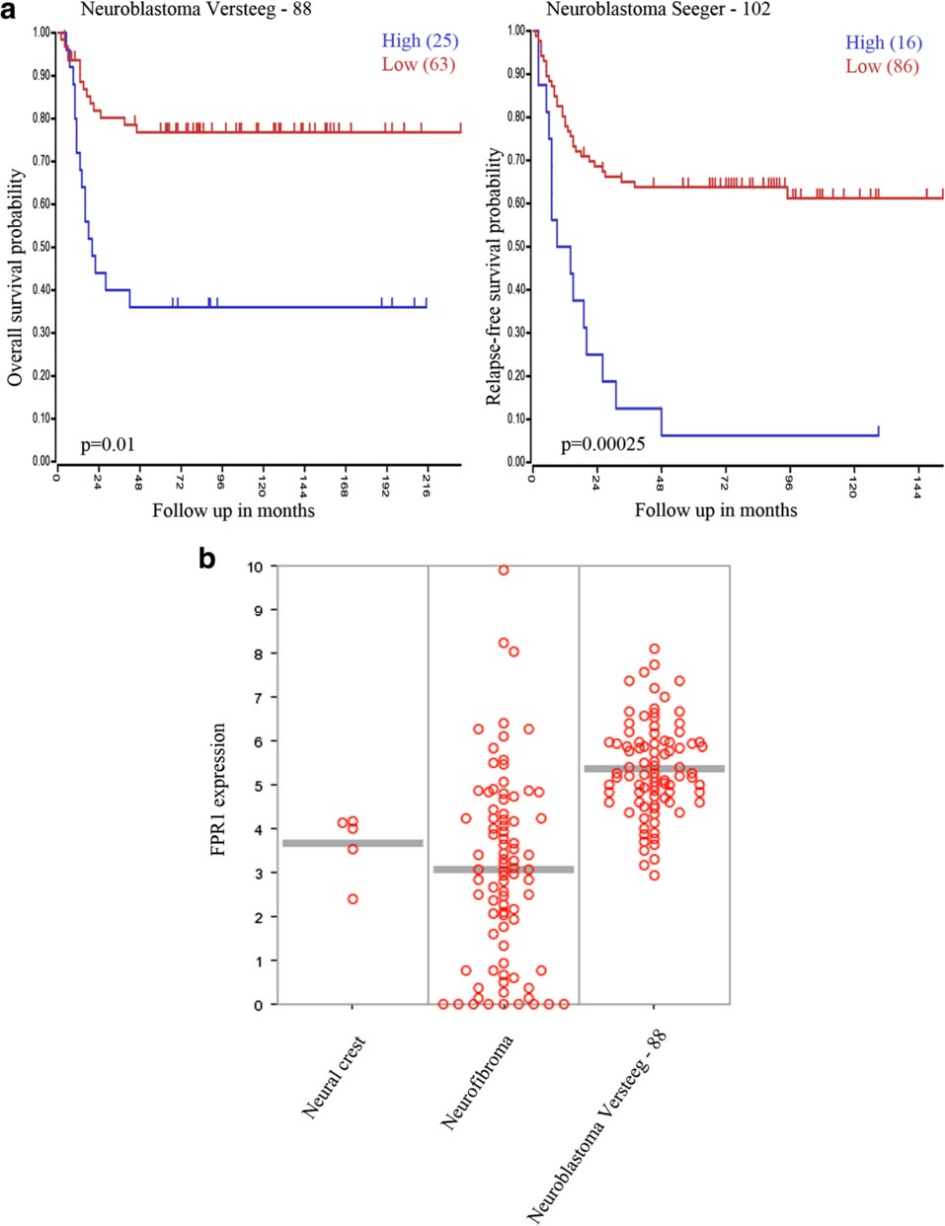
FPR1 gene expression and NB overall survival. **a** High expression of FPR1 in NB is significantly associated with a worse survival. Data were obtained from “R2: Genomics analysis and visualization platform” [http://r2.amc.nl]. Two datasets provided by Versteeg (n = 88) and Seeger (n = 102) were used. **b** Dot plot representing higher expression of FPR1 in Versteeg cohort compared to neural crest tissue and benign neurofibroma. Data were obtained from “R2: Genomics analysis and visualization platform” [http://r2.amc.nl]

Using publicly available expression cohorts, we analyzed the expression levels of FPR1 and the correlation to overall survival in neuroblastoma patients (Fig. 1). FPR1 correlated to poor survival in the majority of expression cohorts analyzed (R2: Genomics Analysis and Visualization Platform: http://r2.amc.nl) [15].

**Fig. 2:**
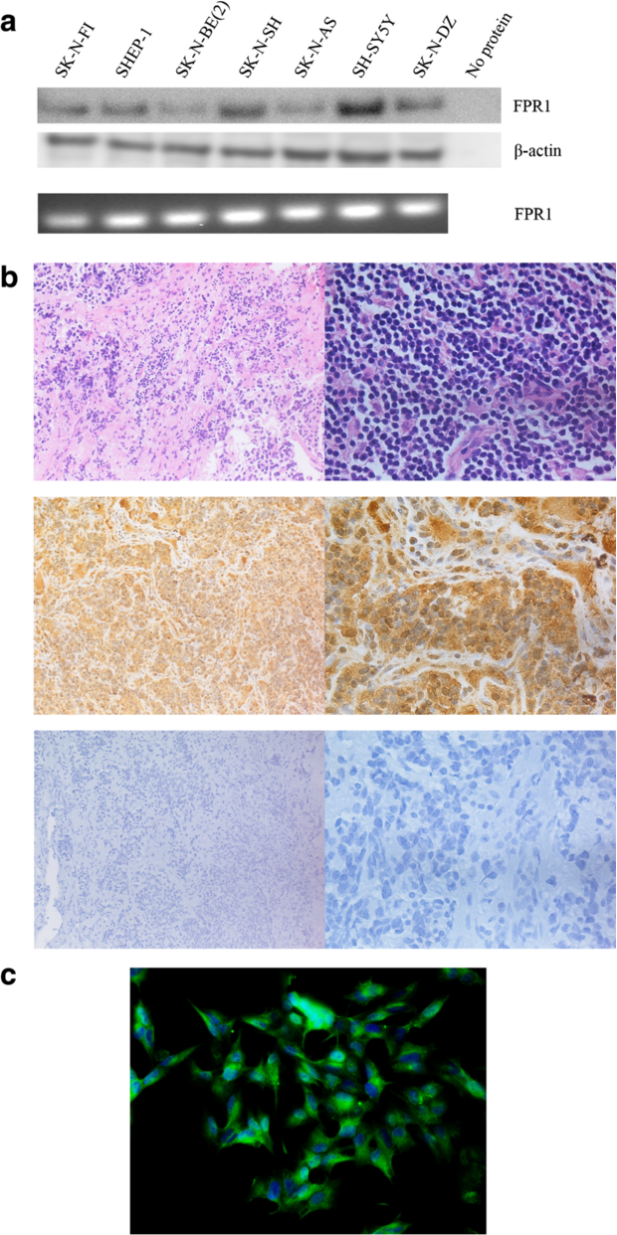
FPR1 is expressed in NB cell lines and primary tumors. **a** Western blotting detected a protein band of approximately 38 kDa corresponding to FPR1 in all NB cells investigated. Additionally, RT-PCR analysis revealed FPR1 expression in 7 NB cell lines. **b** Immunohistochemistry showing specific expression of FPR1 in tumor cells of a primary human neuroblastoma. (upper panel, HE staining, 20x and 60x; middle panel, anti-human FPR1 staining, 20x and 60x; lower panel, isotype control, 20x and 60x) **c** Immunofluorescence staining of FPR1 (green) in SK-N-BE(2) cells

Also, the expression level of FPR1 was analysed in a benign neurofibroma and neural crest revealing a significant lower expression levels compared to neuroblastoma (Fig. 1). Immunohistochemistry and immunofluorescence showed a high expression of FPR1 in neuroblastoma tissue samples and cell lines, respectively (Fig. 2b, c). The staining was localized to the cytoplasm and plasma membrane of tumor cells. Furthermore, all neuroblastoma cell lines analyzed exhibited a significant expression of FPR1, as shown by RT-PCR and western blotting (Fig. 2a).

### FPR1 signaling mobilizes Ca^2+^ and phosphorylation of Erk1/2, Akt and P38

**Fig. 3:**
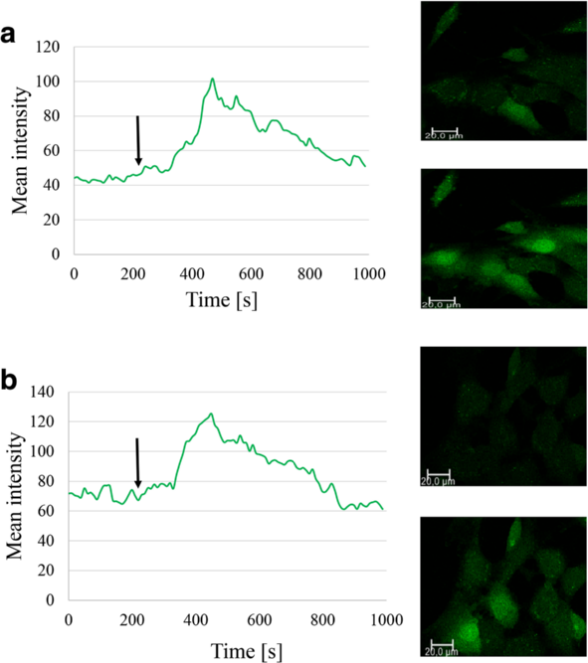
FPR1 stimulation promotes intracellular Ca2+ mobilization. **a** Changes in intracellular calcium level were measured by confocal scanning microscope using green fluorescent calcium-binding dye. After baseline measurement, fMLP was added to the cells’ media and subsequent fluctuations of green fluorescence were monitored. **b** Pretreatment with EDTA. The arrow depicts time of fMLP addition

To investigate the function of FPR1 in neuroblastoma, we stimulated neuroblastoma cells with the FPR1 agonist fMLP. The addition of 10 nM fMLP to SK-N-SH cells induced a rapid increase of intracellular Ca^2+^ (Fig. 3). No differences in Ca^2+^ mobilization were observed when SK-N-SH cells were pre-incubated with the calcium chelator EDTA, thus suggesting an intracellular activation of Ca^2+^.

**Fig. 4:**
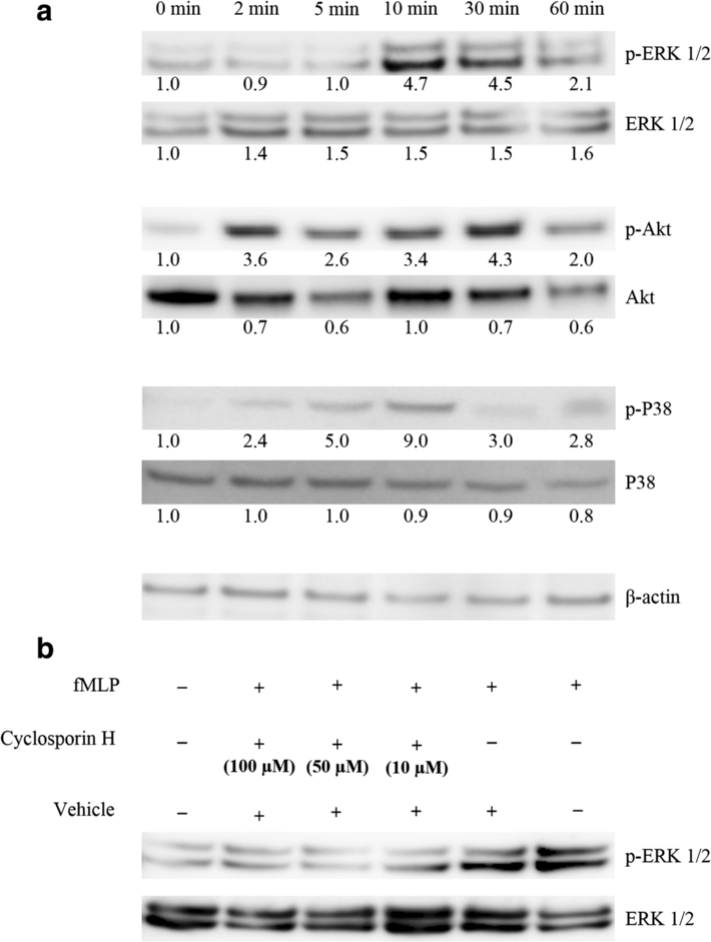
Activation of signal transduction pathways in response to the stimulation of FPR1. **a** Phosphorylation of Erk1/2, Akt and P38-MAPK occurred after stimulation of NB cells with 10 nM of fMLF. Numbers represent densitometric values of bands. **b** Incubation of the cells with Cyclosporine H blocks ERK1/2 phosphorylation

The addition of fMLP (10nM) to serum-starved SH-SY5Y, SK-N-BE(2) and SK-N-AS resulted in a rapid transient phosphorylation of Akt, Erk1/2 and P38 (Fig. 4a). Preincubation of the cells for 15 min with the selective FPR1 antagonist, cyclosporin H, abolished Erk1/2 and Akt phosphorylation induced by the addition of fMLP (Fig. 4b).

### FPR1 promotes neuroblastoma tumorigenesis

**Fig. 5:**
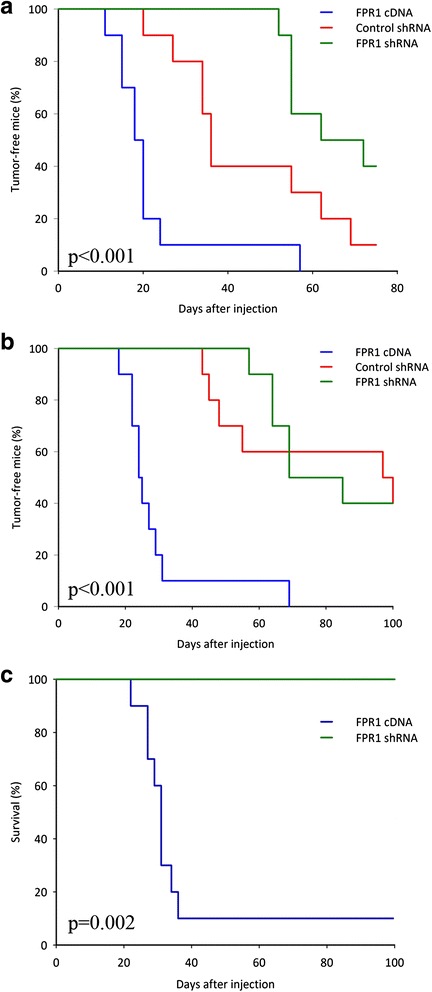
FPR1-dependent tumor development and survival in animal model. Cell lines with different expression level of FPR1 were subcutaneously injected into mice, and tumor growth was recorded. Kaplan-Meier curves represent the time until tumors reached a volume of 0.2 cm^3^ (**a**) and 0.5 cm^3^ (**b**). Survival plot (**c**) shows the difference in survival between animals with high and low expression levels of FPR1 (the criterion for endpoint was a tumor volume ≥ 1 cm^3^)

To assess the role of FPR1 in tumor formation in vivo, we developed a set of SK-N-AS cell clones with either an overexpression or shRNA-mediated knock-down of FPR1. Three groups of nude mice, with 10 animals in each group, were injected with control shRNA, FPR1 shRNA and FPR1 cDNA expressing cells in the hind flanks, and tumor formation was monitored. The time to tumor take, defined as the number of days for a tumor in the animal to reach a volume of 0.2 and 0.5 cm^3^, was prolonged in mice injected with shRNA knock-down cells compared to mice receiving random shRNA cells (CTR) (p < 0.001) (Fig. 5a, b). For SK-N-AS FPR1 knockdown cells, the median time to a tumor volume of 0.2 cm^3^ was more than 50 % longer compared to CTR (55 vs. 36 days) (Fig. 5a). SK-N-AS cells with a forced overexpression of FPR1 developed tumors in nude mice significantly earlier compared to CTR tumors (p < 0.001) (Fig. 5a, b).

## Discussion

N-formyl peptides are cleavage products of bacterial and mitochondrial proteins that can attract leukocytes to sites of infection or tissue damage [16, 17]. Formyl peptide receptor 1 (FPR1) is a cell surface receptor originally described in leukocytes, that binds and is activated by N-formyl peptides, important for the induction of inflammation and immune cell activation. More recently, FPR1 has also been detected in cells of non-myeloid origin such as, smooth muscle cells, lens epithelial cells, fibroblasts, etc. indicating the involvement of the receptor in a wide variety of inflammatory responses [5, 16, 18, 19].

Aberrant expression of FPR1 has been detected in various adult cancers and increased expression of FPR1 in tumors has previously been reported as a negative prognostic factor in patients with gastric cancer, astrocytoma and melanoma [10, 11, 20].

In the present study, we investigated the role of FPR1 in the biology of NB. When using the publicly available “R2: Genomics analysis and visualization platform” [15] with two expression arrays of data from 88 and 102 NB samples, respectively, we discovered that a high FPR1 mRNA expression was correlated with a poor prognosis (Fig. 1). Neurofibroma and neuroblastoma both arise from the neural crest. The level of FPR1 expression was shown to be lower in benign neurofibroma and normal neural crest than in the neuroblastoma, indicating a role of FPR1 in the process of tumorigenesis. Interestingly, FPR1 expression in high-risk tumors, i.e. MYCN-amplified, did not differ from non-amplified MYCN tumors (data not shown) proposing that FPR1 can be used as an independent prognostic marker for overall survival in NB.

By mRNA and western blot analyses, we found that FPR1 was expressed in seven different NB cell lines. Although the different NB cell lines displayed a differential expression of FPR1, no direct relationship between the expression and different genetic aberrations or biological features were observed (Fig. 2a and Table 1)

Recently, Huang et al. showed that glioblastoma cells expressing FPR1 exhibit a highly invasive and malignant phenotype [9, 21]. Additionally, Szczepanek and co-authors demonstrated that FPR1 expression is associated with drug resistance in patients with acute lymphoblastic leukemia [22].

By immunohistochemistry, we analyzed neuroblastoma primary tumors from different biological subsets and clinical stages, and FPR1 expression was detected in all the samples investigated (n = 27) (Fig. 2b), independent of any biological characteristics or clinical stage.

The interaction between FPR1 and its’ ligands triggers a cascade of second messengers through the activation of calcium influx including PI3-kinase, MAP-kinases and NF-kB [23–25]. In order to determine the functional significance of FPR1expression in NB, we incubated the cells with fMLP, a bacterial peptide that induces a significant activation of FPR1 at nanomolar concentrations [5].

Stimulation of neuroblastoma cells with fMLP induced a rapid release of intracellular Ca^2+^ and activation of PI3K/Akt and ERK1/2 signalling (Fig. 3). Similar effects of FPR1 stimulation have recently been described in both normal and tumor cells [18, 20, 26, 27]. Calcium is a ubiquitous second messenger and in cancer it has been implicated in numerous important features of tumorigenesis, including angiogenesis, motility, proliferation and migration [28].

Mitogen-activated protein kinase (MAPK) cascades are key signaling pathways involved in the regulation of normal cell proliferation and survival. These transduction pathways transmit and amplify signals mediated by various growth factors and ligands for G protein-coupled receptors such as fMLP [26].

Activation of the mitogen-activated kinases/extracellular signal-regulated kinases (ERK1/2) has been frequently observed in NB [29].

To further investigate the functional response of the FPR1 activation, we performed a set of Western blots using phospho-specific antibodies. The phosphorylation of Erk 1/2 MAP kinases and P38 MAP kinase was evident upon stimulation with fMLP (Fig. 4a).

There is a large body of evidence that the PI3K/Akt/mTOR pathway plays an important role in the development and progression of NB (reviewed in [30]). The activation of Akt triggers many downstream signaling cascades attributed to tumor growth and survival (reviewed in [31]). We and others have previously shown the specific expression of phosphorylated Akt and mTOR in NB tissue from a variety of clinical and biological stages [29, 32]. In the present work we revealed that the stimulation of NB cells with 10 nM of fMLP results in the phosphorylation of Akt with a peak lying in the interval between 2 and 30 min, which is consistent with previously reported studies with glioblastoma [6, 27] and astrocytoma [20].

After demonstrating that the receptor activation induces signal transduction in NB cells in vitro we wanted to assess the functional significance of FPR1 expression in NB in vivo. We therefore xenografted SK-N-AS cells transfected with lentiviral constructs containing control shRNA, FPR1 shRNA and FPR1 cDNA, respectively.

We observed that animals injected with stable high FPR1 expressing NB cells presented tumors more rapidly compared to the control group of animals and the group with silenced FPR1 (Fig. 5a, b). In addition, nine out of ten animals with a high expression of the receptor reached the experimental endpoint (tumor volume ≥ 1 cm^3^) by day 36 after injection, while none of the animals from the other groups reached the endpoint by day 100 after injection (Fig. 5c).

Our data are in line with results reported in a human glioblastoma model, in which FPR1 expressing U-87 cells that were either silenced by siRNA or inhibited by FPR1 antagonist exhibited a delayed tumorigenicity in vitro [6, 20, 33].

How elevated FPR1 expression in NB may contribute to tumorigenicity is not known. The induction of FPR1 may trigger a wide variety of effects attributed to tumor growth such as increased vascular permeability and angiogenesis, chemotaxis, cell adhesion, as well as cell survival and proliferation [6, 8]. Besides fMLP, ligands for FPR1 also include non-microbial endogenous host-derived peptides such as mitochondrial formylated peptides fMMYALF, fMLKLIV and fMFADRW, Annexin A1 and Cathepsin G [5, 34, 35], that may be released by necrotic cells within the tumor microenvironment.

Recently, several reports have shown that tumor cells have co-opted some of the signaling molecules of the innate immune system, such as arachidonic metabolites [36], chemokines [37], alarmins [38] and their receptors for invasion, migration and metastasis [21]. We assume that FPR1 expressed by NB cells may be activated by formylated peptides released from the mitochondria or by other compartments of necrotic tumor cells, locally acting as trophic factors which promote FPR1-driven inflammatory signaling pathways, amplified growth and invasiveness. Hence, the recent development of FPR1 antagonists modulating the inflammatory processes makes FPR1 a promising target for adjuvant NB therapy [39].

## Conclusions

In summary, we have shown that FPR1 is expressed in human NB tissue and that the receptor is functionally active in human NB cells. Furthermore, the inhibition of FPR1 activity in NB cells resulted in a decreased tumor growth in a xenograft model while overexpression resulted in an increased tumor load, suggesting a role for FPR1 in the development of an aggressive phenotype.

Our results are supported by microarray survival data from human tumor samples, demonstrating that a high FPR1 expression is associated with significantly lowered overall patient survival.

Pharmacological intervention that targets FPR1 in NB may become an interesting adjuvant therapy for children with NB but further preclinical studies are warranted.

## Abbreviations

cDNA: complementary DNA; CTR: control; FBS: fetal bovine serum; fMLP:N-Formyl-L-methionyl-L-leucyl-L-phenylalanine; FPR1: formyl peptide receptor 1; HHBS: Hank’s Balanced Salt Solution; mRNA: messenger RNA; NB: neuroblastoma; PBS: phosphate-buffered saline; RFP: red fluorescent protein; RT-PCR: reverse transcription polymerase chain reaction; shRNA: short hairpin RNA.

## Additional files

Additional file 1: Figure S1. Complete WB membranes and PCR gels images for Fig. 2. Lower gel represents no RT controls for all cell lines (lanes 1–7) and no template control (lane 8). (TIF 2142 kb) Additional file 2: Figure S2. Complete WB membranes for Fig. 4. (TIF 926 kb) Additional file 3: Figure S3. Complete WB membranes for Fig. 4. (TIF 1188 kb)
