# Supplementary figures and images for: The role of formyl peptide receptor 1 (FPR1) in neuroblastoma tumorigenesis
Source: BMC Cancer. 2016 Jul 18;16:490. doi: 10.1186/s12885-016-2545-1 (PMC4950242; doi:10.1186/s12885-016-2545-1)

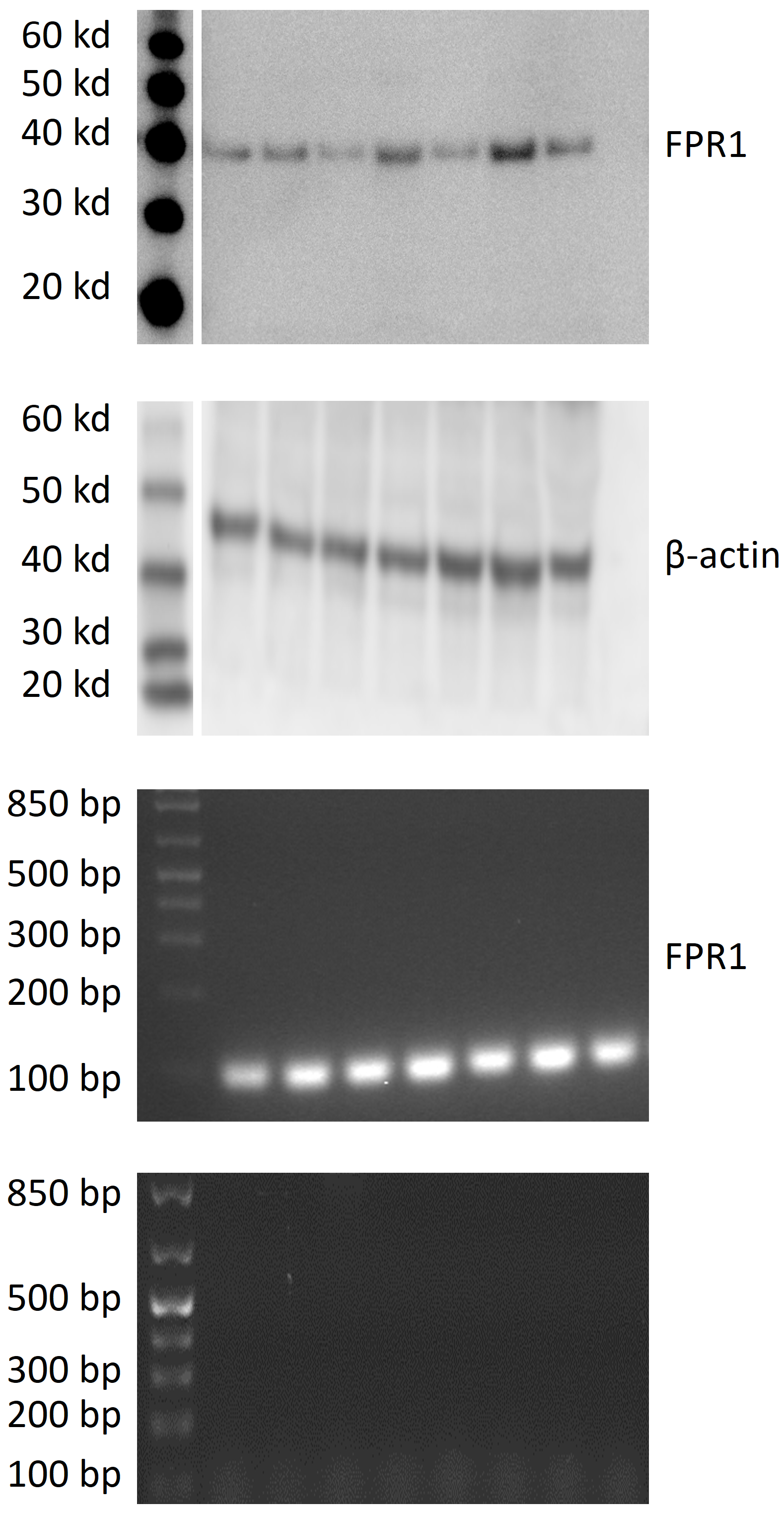

Supplement: Additional file 1: Figure S1. — Complete WB membranes and PCR gels images for Fig. 2. Lower gel represents no RT controls for all cell lines (lanes 1–7) and no template control (lane 8). (TIF 2142 kb) [file 12885_2016_2545_MOESM1_ESM.tif]

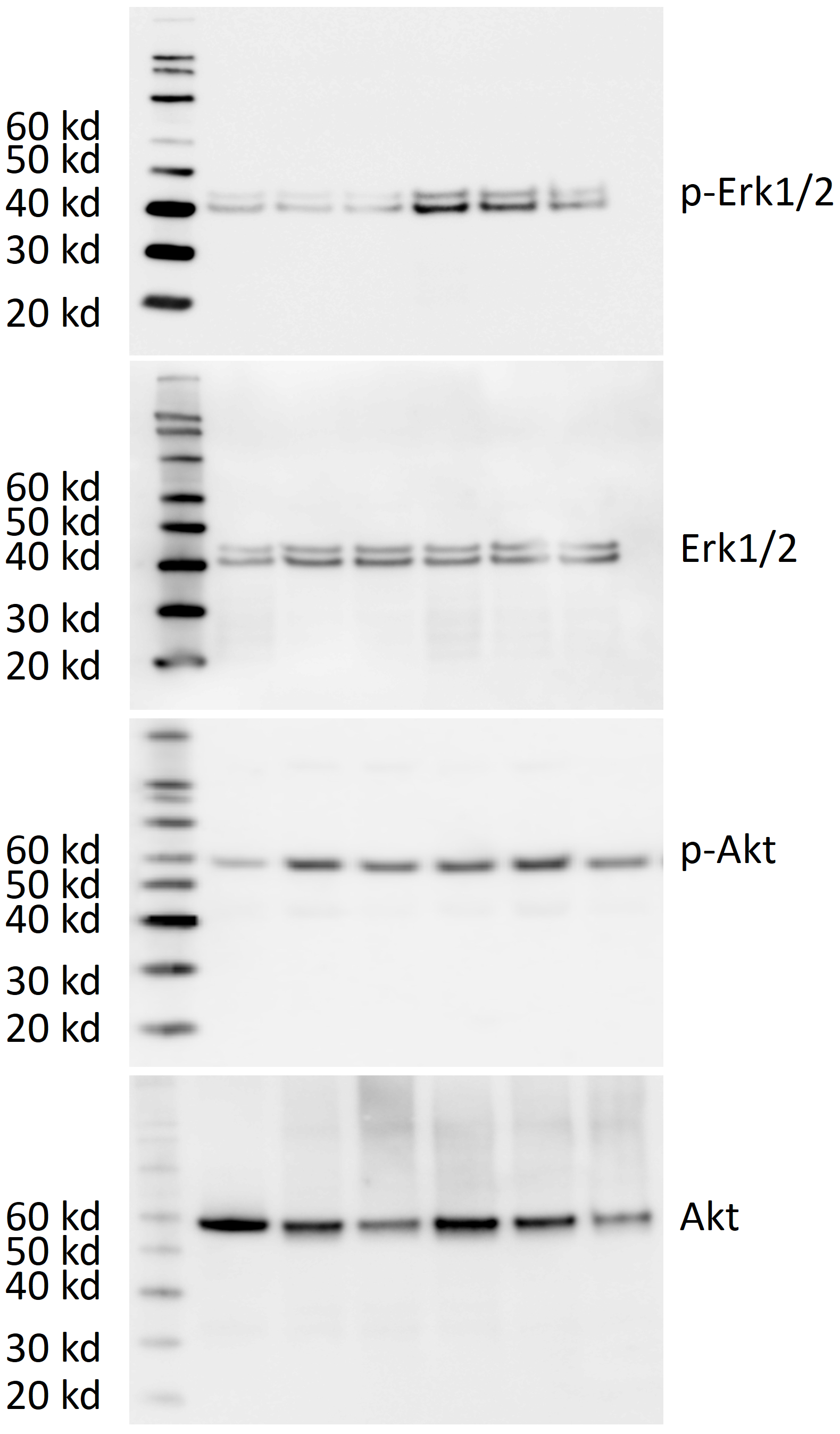

Supplement: Additional file 2: Figure S2. — Complete WB membranes for Fig. 4. (TIF 926 kb) [file 12885_2016_2545_MOESM2_ESM.tif]

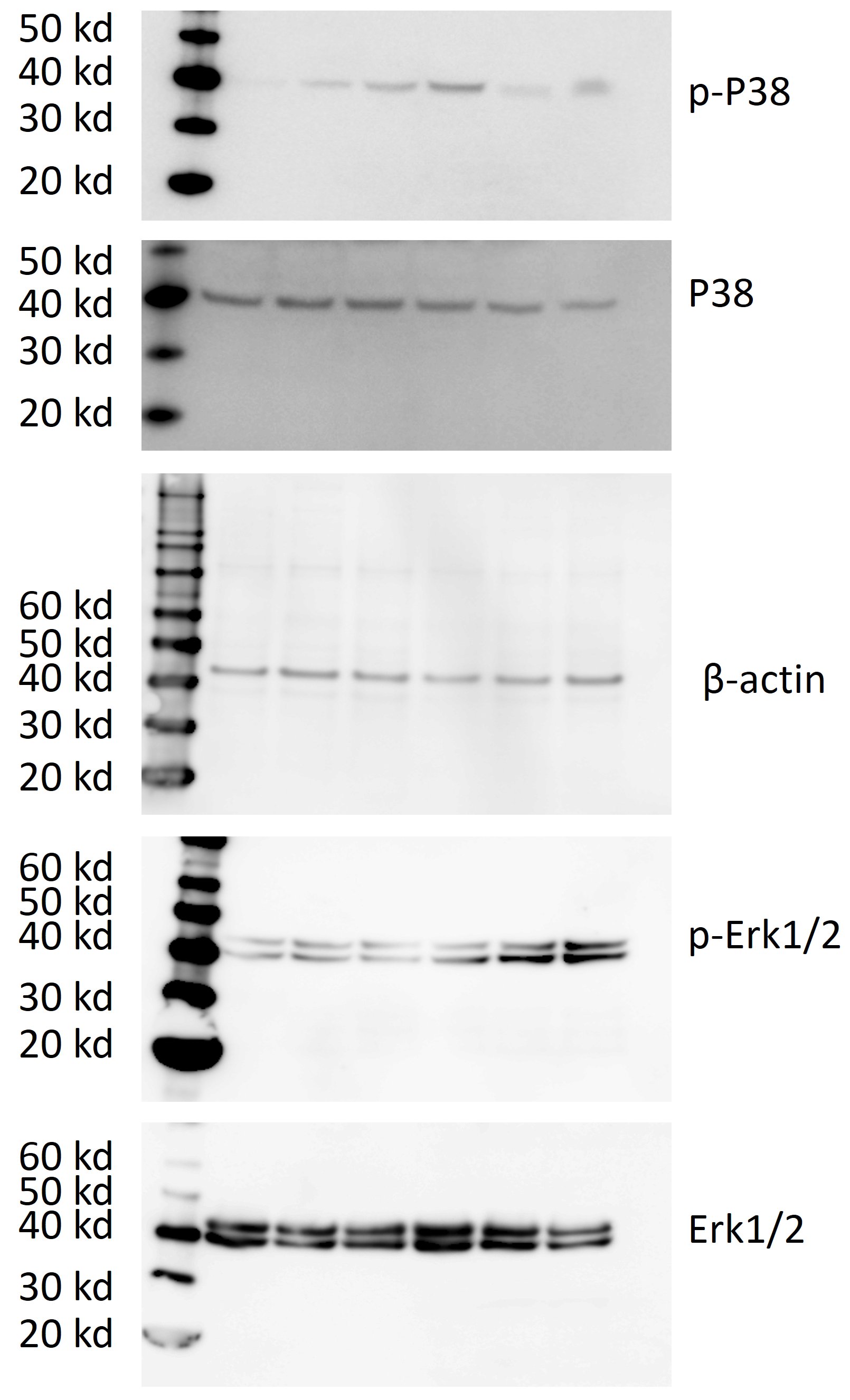

Supplement: Additional file 3: Figure S3. — Complete WB membranes for Fig. 4. (TIF 1188 kb) [file 12885_2016_2545_MOESM3_ESM.tif]
